# Mechanical properties of epithelial cells in domes investigated using atomic force microscopy

**DOI:** 10.3389/fcell.2023.1245296

**Published:** 2023-11-15

**Authors:** Kenta Shigemura, Kaori Kuribayashi-Shigetomi, Ryosuke Tanaka, Haruka Yamasaki, Takaharu Okajima

**Affiliations:** ^1^ Graduate School of Information Science and Technology, Hokkaido University, Sapporo, Japan; ^2^ Institute for the Advancement of Higher Education, Hokkaido University, Sapporo, Japan; ^3^ Faculty of Information Science and Technology, Hokkaido University, Sapporo, Japan

**Keywords:** epithelial dome, atomic force microscopy, cell mechanics, Young’s modulus, tension

## Abstract

As epithelial cells *in vitro* reach a highly confluent state, the cells often form a microscale dome-like architecture that encloses a fluid-filled lumen. The domes are stabilized by mechanical stress and luminal pressure. However, the mechanical properties of cells that form epithelial domes remain poorly characterized at the single-cell level. In this study, we used atomic force microscopy (AFM) to measure the mechanical properties of cells forming epithelial domes. AFM showed that the apparent Young’s modulus of cells in domes was significantly higher when compared with that in the surrounding monolayer. AFM also showed that the stiffness and tension of cells in domes were positively correlated with the apical cell area, depending on the degree of cell stretching. This correlation disappeared when actin filaments were depolymerized or when the ATPase activity of myosin II was inhibited, which often led to a large fluctuation in dome formation. The results indicated that heterogeneous actomyosin structures organized by stretching single cells played a crucial role in stabilizing dome formation. Our findings provide new insights into the mechanical properties of three-dimensional deformable tissue explored using AFM at the single-cell level.

## Introduction

The epithelial dome is a fundamental structure for various physiological functions, including morphogenesis, transport, and secretion. As epithelial cell monolayers *in vitro* are cultured to a highly confluent stage, monolayers often exhibit spontaneous formation of microscale dome-like architecture that encloses the fluid-filled lumen between the monolayer and the culture substrate (Leighton et al., 1969; Leighton et al., 1970; McGrath and Blair, 1970; McGrath, 1975; Lever, 1979a; Lever, 1981). Domes formed in epithelial monolayers have been widely used as tissue models to investigate the mechanisms of luminal formation (Latorre et al., 2018) and physiological maintenance (Nicolas and Lievin-Le Moal, 2015).

Domes are formed from Madin–Darby canine kidney (MDCK) cell lines (Leighton et al., 1969; Leighton et al., 1970; Lever, 1979a; Lever, 1981; Thomas et al., 1982), and other types of fluid-transporting epithelial cells (McGrath and Blair, 1970; Lever, 1979b; Goodman and Crandall, 1982; Mason et al., 1982; Goodman et al., 1984; Fantini et al., 1986; Zucchi et al., 2002; Cattaneo et al., 2011). Dome formation is triggered by a fluid flow pumped through cells in the apicobasal direction, which pressurizes the interstitial space between the cell monolayer and the culture substrate (Leighton et al., 1969; Tanner et al., 1983; Ishida-Ishihara et al., 2020). During dome formation, the number of cells remains almost constant (Latorre et al., 2018). This process stretches the cells and the dome tension balances the osmotic pressure, while obeying Laplace’s law (Latorre et al., 2018). A recent study on traction force measurements of domes and theoretical modeling of epithelial cells in domes revealed that the epithelial dome is regulated via cellular deformation, mechanical stress, and luminal pressure (Latorre et al., 2018). However, the mechanical properties of cells forming domes remain poorly characterized.

In this study, we directly measured the mechanical properties of epithelial MDCK domes using force–indentation atomic force microscopy (AFM), which has been used extensively to explore the mechanical properties of two-dimensional cell monolayers (Hoh and Schoenenberger, 1994; A-Hassan et al., 1998; Brückner and Janshoff, 2015; Fujii et al., 2019; Nehls et al., 2019). AFM showed that the cell stiffness in domes was significantly increased compared with that in the surrounding monolayer. AFM also revealed that the stiffness and tension of domes were positively correlated with the apical cell area. This correlation disappeared as actin filaments were depolymerized or the ATPase activity of nonmuscle myosin II was inhibited, suggesting that heterogeneous actomyosin structures organized via cell stretching during dome formation played a crucial role in stabilizing dome formation.

## Materials and methods

### Micropatterned substrate

A micropatterned glass substrate with circular regions of 95 μm in diameter was fabricated to control the basal area of the domes. Fibronectins (Sigma‒Aldrich, St. Louis, MO, United States) were used to coat the flat glass substrate outside the circular regions. Within the circular regions, a 2-methacryloyloxyethyl phosphorylcholine (MPC) polymer (Lipidure®-CR3001; Nippon Oil & Fats, Tokyo, Japan) was chemically bound to the glass substrate surface via silane coupling, preventing cell attachment. Then, aluminum was deposited on the glass substrate and etched to form circular patterns using a standard photolithographic technique with a positive photoresist (MICROPOSIT S1818; Dow, MI, United States). The photoresist was removed, the substrate was cleaned by plasma etching, an MPC solution was spun on the substrate, and the substrate was dried. The aluminum film was lifted off to produce MPC-coated circular micropatterns on the glass substrate.

### Cell sample

**FIGURE 1:**
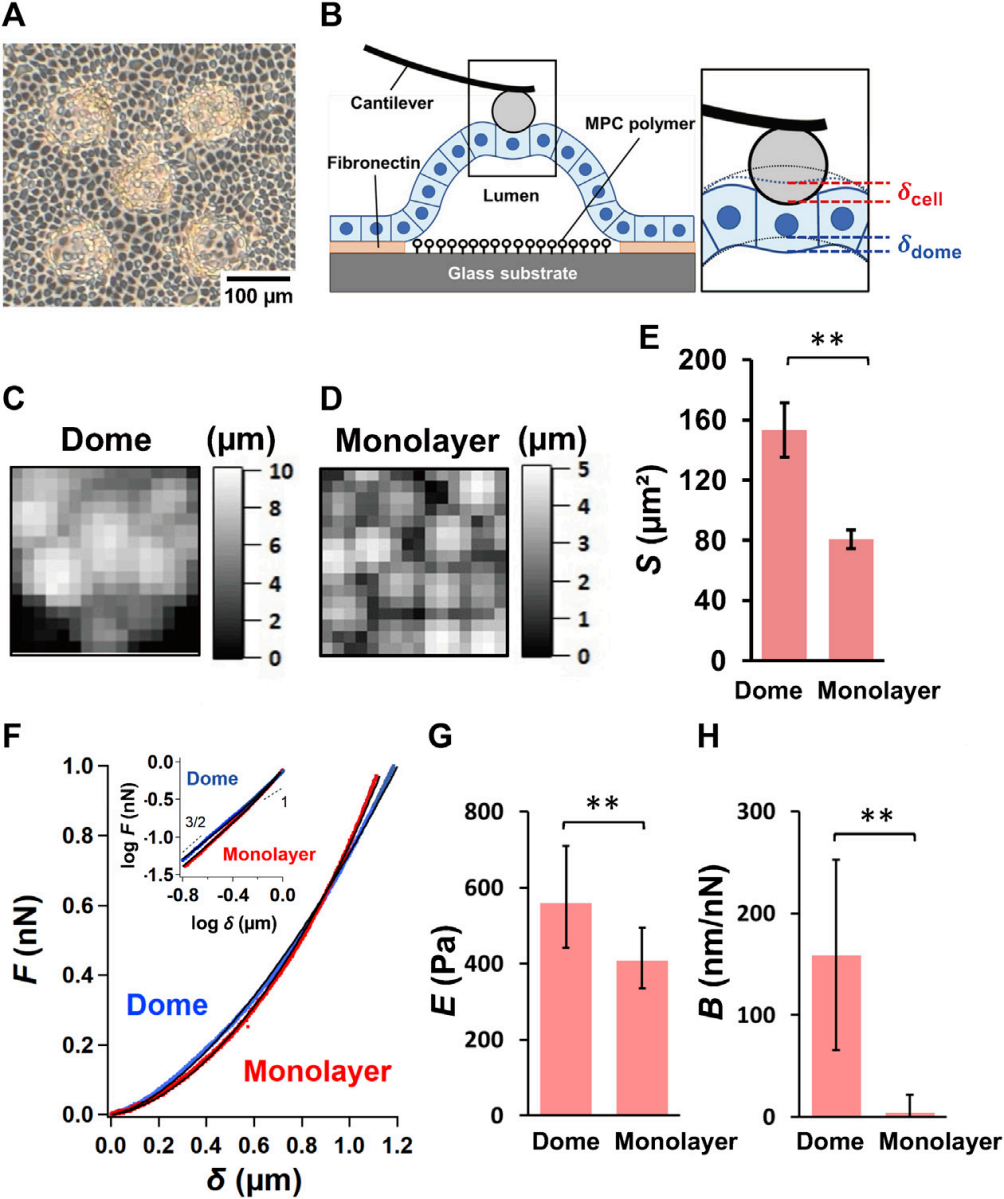
Characterization of epithelial MDCK domes and monolayers. **(A)** A representative optical microscopic image of epithelial MDCK domes formed on the micropatterned substrate. **(B)** Schematic representation of the AFM measurements of the epithelial dome. The inset shows the magnification of the indentation region located between the AFM probe and the dome. The dotted lines represent the apical morphology of the dome before indentation (black) and the apical deformation of the dome caused by the basal deformation of the dome (blue). Representative AFM height images (48 × 48 mm^2^) of the region around the top of the dome **(C)** and in monolayer **(D)** samples. **(E)** Quantification of the apical cell area, *S*, of the dome (*n* = 32 cells from 9 domes, 6 independent experiments (dishes), with 5 cell culture passages) and monolayer (*n* = 24 cells from 6 monolayers, 5 dishes, with 4 cell culture passages). **(F)** Average force–indentation curves measured in a region within cells around the top of domes (blue) and in monolayers (red). The inset shows the curves presented using the log–log scale. The solid lines represent the fitted curves of Eq. 2. The quantification of log *E* **(G)** and *B* **(H)** in domes (*n* = 14 domes, from 4 dishes, with 4 cell culture passages) and monolayers (*n* = 8 monolayers from 4 dishes, with 4 cell culture passages). Data are presented as the arithmetic mean ± standard deviation. *p < 0.05 and **p < 0.01.

MDCK cells were cultured at 37°C and 5% CO_2_ in a minimal essential medium (Sigma‒Aldrich) with 10% fetal bovine serum, 1% penicillin/streptomycin, and 1% nonessential amino acids (Sigma‒Aldrich). The cells were trypsinized using 0.25% trypsin/EDTA (Sigma‒Aldrich) and seeded at an initial concentration of 1.0 × 10^6^ cells on the patterned glass substrate. The cell sample was cultured for ∼3 days until an epithelial dome was formed (Figure 1A). The cultured MDCK cell monolayer attained a highly confluent state, after which cell migration halted entirely (Fujii et al., 2019).

### AFM measurement

We used a customized atomic force microscope attached to an upright optical microscope (Eclipse FN1; Nikon, Tokyo, Japan), which was similar to the experimental setup used in previous studies (Fujii et al., 2019; Tanaka et al., 2020; Fujii et al., 2021). The deflection of a rectangular cantilever (BioLever mini, BL-AC40TSC2; Olympus, Tokyo, Japan) was detected through a water-immersed objective lens (CFI Plan fluor 10xW; Nikon). A silica bead with a radius *R* of ∼5 μm (Funakoshi, Tokyo, Japan) was attached with epoxy resin to the apex of the cantilever tip to achieve a well-defined contact geometry (Cai et al., 2013; Cai et al., 2017; Fujii et al., 2019). The loading force was determined using Hooke’s law (multiplying the cantilever deflection by the spring constant) and calibrated using a thermal fluctuation method (Hutter and Bechhoefer, 1993).

In the force-mapping measurements, the cells were indented at a maximum loading force of ∼1.0 nN. The loading force *F* caused deformation of cells and dome (Figure 1B). For cell deformation, the apparent Young’s modulus of the cell, *E*, was estimated from the Hertzian contact model (Schillers et al., 2017) and expressed as follows:

$$F=\frac{4}{3}\frac{ER^{1/2}}{1-\nu^{2}}\delta_{\text{cell}}^{3/2},$$ (1)

where *δ*_cell_ was the indentation depth of the cell (Figure 1B) and *ν* was Poisson’s ratio of the cell (assumed here to be 0.5, which corresponded to a perfectly incompressible material). For dome deformation, *F* involved the tension and isotropic internal pressure supporting the dome (Salbreux et al., 2012; Latorre et al., 2018; Chan and Hiiragi, 2020; Gómez-González et al., 2020). According to a model that comprises spherical-shaped soft materials placed on a substrate and obeys Laplace’s law (Cartagena-Rivera et al., 2016), the force compressed by an indenter at the top of the material is approximately proportional to the indentation depth. Thus, we assumed that *F* was proportional to the indentation depth of the dome, *δ*_dome_ (Figure 1B). The measured overall indentation depth *δ* was the sum of the indentation depths of the cell and dome (*δ* = *δ*_cell_ + *δ*_dome_), which was analogous to soft cells on deformable substrates (Rheinlaender et al., 2020). Thus, we determined the relationship between *δ* and *F* via the following equation:

$$\delta \left(F\right)=AF^{2/3}+BF,$$ (2)

where $A{=\left({16ER}^{1/2}/9\right)}^{-2/3}$ (Rheinlaender et al., 2020) and *B* was the factor relating to the intercellular tension and osmotic pressure (Salbreux et al., 2012; Latorre et al., 2018; Chan and Hiiragi, 2020; Gómez-González et al., 2020); here, an increase in *B* corresponded to a decrease in tension and osmotic pressure.

For AFM measurements, the medium was replaced with a CO_2_-independent medium (Gibco, Grand Island, NY, United States), and the temperature was maintained at 33°C during measurements. To avoid the effect of the surface tilt of cells in domes (Fujii and Okajima, 2019), force curve mapping measurements were performed around the top of domes. The scanning range was 48 × 48 μm^2^ for a spacing of 3 μm, which resulted in a low spatial resolution for the requirement of achieving a short measurement time because domes often fluctuate slowly (Latorre et al., 2018). After removing the virtual deflection of the force curves and determining the baseline in the noncontact region, the initial contact point was set to the data point of the approach curve that contacted with the baseline of the force curve when the data point was changed from the trigger force (∼1 nN) to zero. During the change in the position of the contact point in the region close to the initial contact point, the force curves were fitted to Eq. 2, and the solution providing the lowest norm of residuals was chosen as the final contact point.

Apical cell area *S* was determined by defining the cell-cell boundary to be positioned at the local minimum of the height images (Fujii et al., 2019). We estimated *E* and *B* by averaging them from the force–indentation curves (typically 4‒9 curves), which are measured around the center of the cells (that is, the cytoplasmic regions). Single-cell mechanics studies have revealed that elastic terms such as *E* and storage modulus approximately follow a log-normal distribution (Fabry et al., 2001; Balland et al., 2006; Cai et al., 2013). Thus, we plotted log *E* as arithmetic mean ± standard deviation. The cell samples were treated with 1 μM latrunculin A (Sigma‒Aldrich) to inhibit actin filament polymerization. The ATPase activity of nonmuscle myosin II was inhibited by treatment with 50 μM blebbistatin (Sigma‒Aldrich). The AFM experiments of treated samples were performed 10 min after inhibitor treatment.

### Fluorescence live imaging

The fixation of cells with 4% paraformaldehyde caused the domes to contraction. Therefore, we used Sara Fluor™ 497 actin probe (Goryo Chemical, Inc., Japan) to stain actin filaments of epithelial cells in domes and monolayers. The samples were incubated for 30 min with a 100 nM probe-containing medium and then were replaced with a CO_2_-independent medium. We used a laser-scanning confocal microscope (C1, Nikon, Japan) with a ×20 objective (Plan Apo, 0.75NA, Nikon) to obtain z-stack images between the apical and basal surfaces of domes and monolayers at 2 μm z-intervals. The fluorescence intensities of actin filaments in an area (30 μm × 30 µm) around the top of the domes and in monolayers were added and averaged using stacked images between apical and basal surfaces of cells with ImageJ software.

## Statistics

**FIGURE 2:**
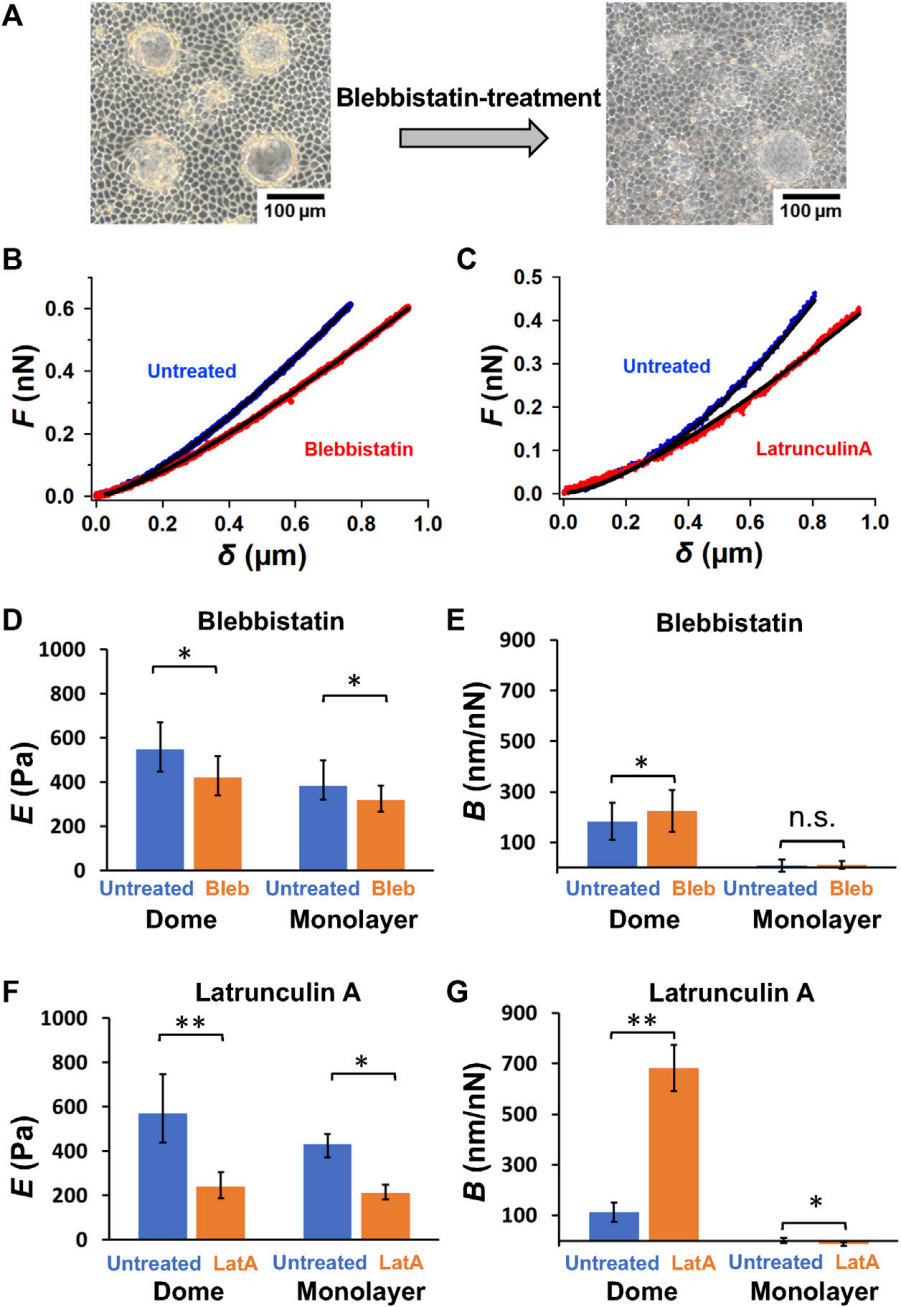
Actin filament network and myosin II activity play a crucial role in sustaining dome formation. **(A)** Representative images of epithelial domes before and after blebbistatin treatment for 2 h. Representative force–indentation curves of untreated and blebbistatin-treated cells **(B)**, as well as untreated and latrunculin A-treated cells **(C)**, are shown. The solid lines represent the fitted curves of Eq. 2. Quantification of log *E* **(D)** and *B* **(E)** in blebbistatin-treated cells in domes (*n* = 7 domes, from 4 dishes, with 2 cell culture passages) and monolayers (*n* = 4 monolayers, from 4 dishes, with 2 cell culture passages), and log *E* **(F)** and *B* **(G)** in latrunculin A-treated cells in domes (*n* = 7 domes, from 4 dishes, with 2 cell culture passages) and monolayers (*n* = 4 monolayers, from 4 dishes, with 2 cell culture passages). Data are presented as the arithmetic mean ± standard deviation. *p < 0.05 and **p < 0.01.

**FIGURE 3:**
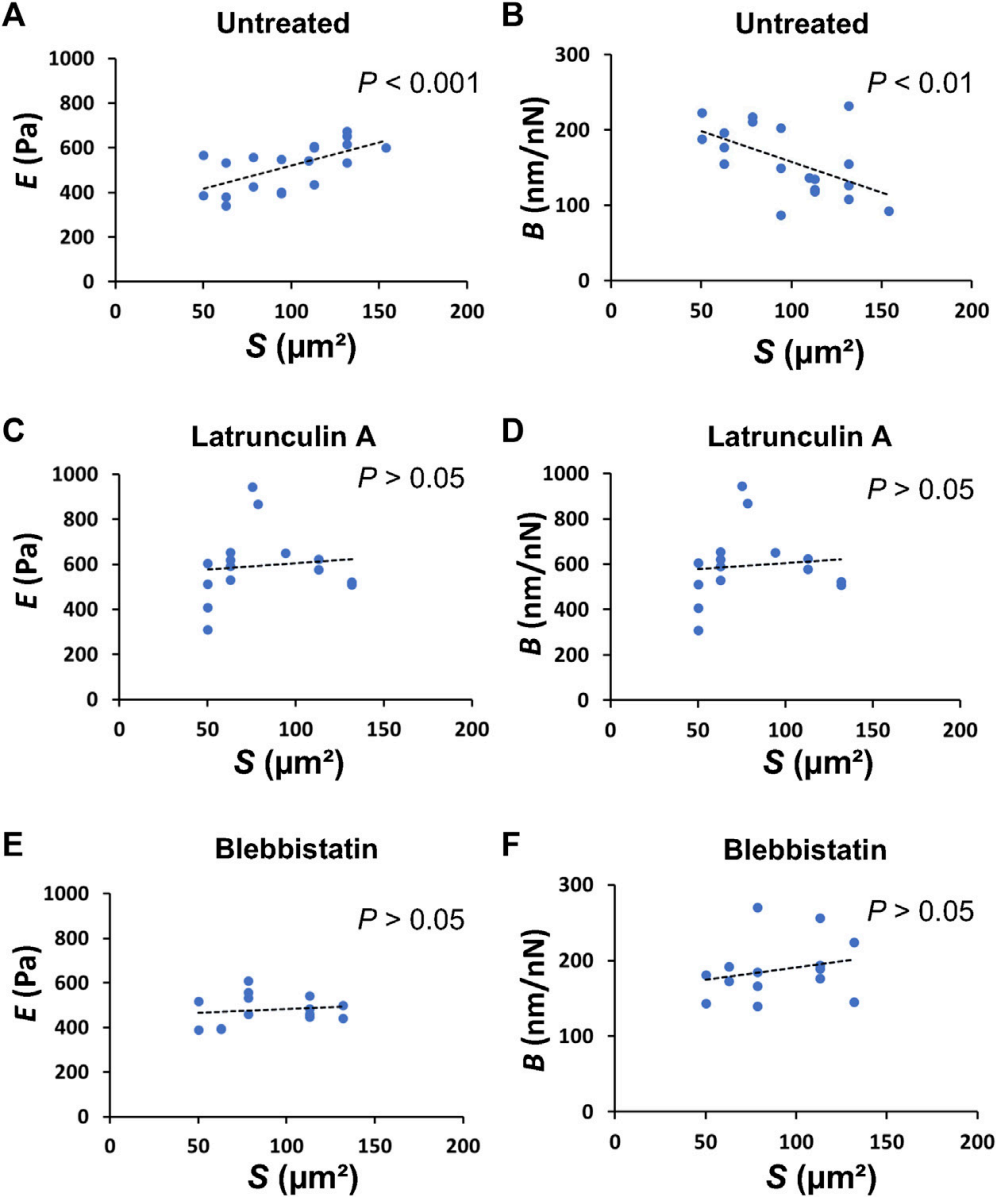
The apical cell area in domes directly correlates to the mechanical properties of the cells. Plots of *E versus S* in untreated [(**A)**; *n* = 19 cells from 5 domes, 5 dishes, with 2 passages; *r* = 0.6161; *p* = 4.975 × 10^−3^], latrunculin A-treated [**(C)**; *n* = 15 cells from 4 domes, 4 dishes, with 2 passages; *r* = 0.02186; *p* = 0.9384], and blebbistatin-treated [**(E)**, *n* = 14 cells from 4 domes; *r* = 0.1801; *p* = 0.5379] cells in domes. Plots of *B versus S* in untreated [**(B)**, *n* = 19 cells from 5 domes, 5 dishes, with 2 passages; *r* = −0.5545; *p* = 1.375 × 10^−2^], latrunculin A-treated [**(D)**, *n* = 15 cells from 4 domes, 4 dishes, with 2 passages; *r* = 0.2496; *p* = 0.3696], and blebbistatin-treated [**(F)**, *n* = 14 cells from 4 domes, 4 dishes, with 2 passages; *r* = 0.2377; *p* = 0.4132] cells in domes.

**FIGURE 4:**
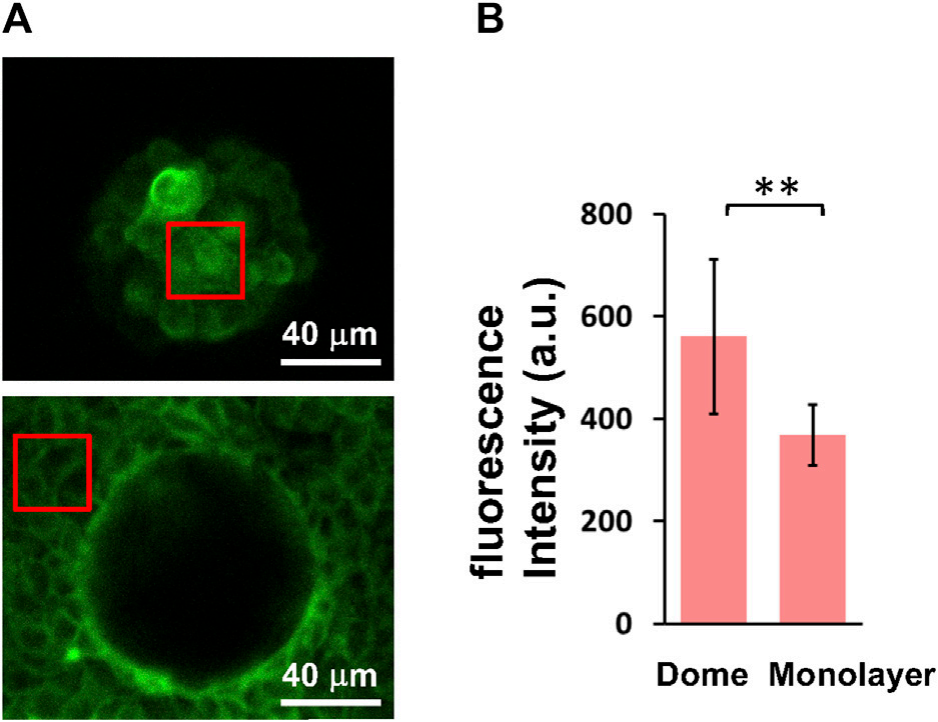
Cellular actin filament density is increased in domes compared to monolayers. **(A)** Representative fluorescence images of actin filaments in cells at the top of domes (upper) and in monolayers (lower) were obtained using a confocal microscope. **(B)** Quantification of fluorescence intensity of cells in domes (*n* = 6 domes, from 2 dishes, with 1 passage) and monolayers (*n* = 6 monolayers, from 2 dishes, with 1 passage). The red boxes represent examples of regions of interest used for quantification. Data are presented as the arithmetic mean ± standard deviation. **p < 0.01.

First, to estimate the normality of data, we conducted statistical analysis using the Shapiro-Wilk test and then, to compare the data before and after treatment with latrunculin A and blebbistatin (Figures 2D−G), we performed either a two-tailed paired Student’s *t*-test or the Wilcoxon signed-rank test. The Welch’s *t*-test and Mann-Whitney *U* test were used for comparing other data (Figures 1E, G, H and Figure 4B). Based on the normality of the data, as determined using the Shapiro-Wilk test, the Pearson or Spearman correlation coefficient (*r*) test was used to correlate the two data sets (Figure 3). The *p*-values <0.05 were considered to be significant. Experimental numbers, such as individual cell numbers, dishes, cell culture passages, and domes or monolayers, are indicated in the figure legends.

## Results

### Mechanical properties of cell domes and monolayers

The force–indentation AFM images depicting the relative height were obtained around the top of the domes (Figure 1C) and in monolayers (Figure 1D), thereby enabling identification of individual cells in these cell systems. The apical cell area, *S*, observed using AFM, significantly increased in the domes when compared to that in the monolayers (Figure 1E), indicating that the cells in the domes were highly stretched.

The force–indentation curves measured around the central region of cells in monolayers showed a nonlinear function and displayed a single power–law function with an exponent of 3/2, which follows the conventional Hertz contact model in Eq. 1 (Figure 1F). Moreover, the force–indentation curve measured around the central region of cells around the top of domes seemed to follow a power–law function with an exponent of 3/2 for small indentations, and the exponent tended to decrease for large indentations (Figure 1F). We quantified *E* and *B* from force–indentation curves using Eq. 2 and found that *E* of cells in the dome was significantly greater than that in the monolayer (Figure 1G). *B* was almost zero in the monolayer, but *B* in the dome was significantly larger than that in the monolayer (Figure 1H). According to the model (Cartagena-Rivera et al., 2016), tension σ in the dome equals 1/(π*B*); that is, σ was 1.3–4.5 mN/m when *B* = 70–250 nN/nm (Figure 1F). The dome tension estimated by using AFM was in good agreement with that measured on a soft substrate using traction force microscopy (Latorre et al., 2018), thus supporting that *B* is an indicator associated with deformation of the dome indented by the AFM probe.

### Chemical modification

To understand the effect of actomyosin networks on the dome mechanics, we investigated changes in dome shape after ATPase activity of nonmuscle myosin II was inhibited with blebbistatin, which tends to reduce the internal tension in domes. The blebbistatin-treated domes exhibited unstable dynamics, often contracting within a few hours (Figure 2A). We observed that the contraction speed varied greatly between the treated domes. Thus, by selecting the domes that slowly changed their shape in the initial stage, we analyzed force-indentation curves of cells around the center of domes before and after the blebbistatin treatment to estimate the mechanical properties of cells (Figure 2B). We found that blebbistatin treatment caused a significant reduction in *E* in both dome and monolayer samples (Figure 2D). This indicated that *E* measured using AFM was strongly associated with the activity of myosin II. Blebbistatin treatment also showed a significant increase in *B* (Figure 2E) in domes, indicating that the dome largely reduced the tension and balanced the osmotic pressure when the activity of myosin II was inhibited. In contrast, *B* in monolayers remained at zero (Figure 2D), thereby agreeing with our model in which *B* is associated with the deformation of the dome indented by the AFM probe (Figure 1B).

We also observed dome contraction when actin filaments of cells in domes were depolymerized with latrunculin A. We measured force-indentation curves before and after the latrunculin A treatment (Figure 2C) and found that *E* (Figure 2F) and *B* (Figure 2G) in latrunculin A-treated dome samples exhibited the same behavior as in blebbistatin-treated samples (Figures 2D, E). These results indicated that the network structure of actin filaments and the activity of myosin II were crucial to sustaining dome formation.

### Mechanical properties of single cells

To understand how single cells that form domes change their mechanical properties, we investigated the relationship between the mechanical properties of single cells in domes and cell shape. The plot of *E versus S* of single cells in domes showed a significant positive correlation with the Pearson correlation coefficient *r* = 0.6161 and *p* = 4.975 × 10^−3^ (Figure 3A), while exhibiting a significant negative correlation between *B* and *S* (*r* = −0.5545, *p* = 1.375 × 10^−2^) (Figure 3B). Importantly, these correlations disappeared when cells in the dome were treated with latrunculin A (Figures 3C, D) or blebbistatin (Figures 3E, F), indicating that the cell shape in domes was highly associated with the mechanical properties of the cells.

To investigate the formation of actin filaments in cells that form domes and monolayers, we observed live images of actin filaments-stained cells with a laser-scanning confocal microscope (Figure 4A). The fluorescence in dome cells appears to be relatively brighter in the cortex compared to that in the cell-cell boundaries. Our results showed that the fluorescence intensity of cells in domes was significantly higher than that in monolayers (Figure 4B), indicating that the actin filament density in cell cortical regions was increased in domes compared with that in monolayers.

## Discussion

**FIGURE 5:**
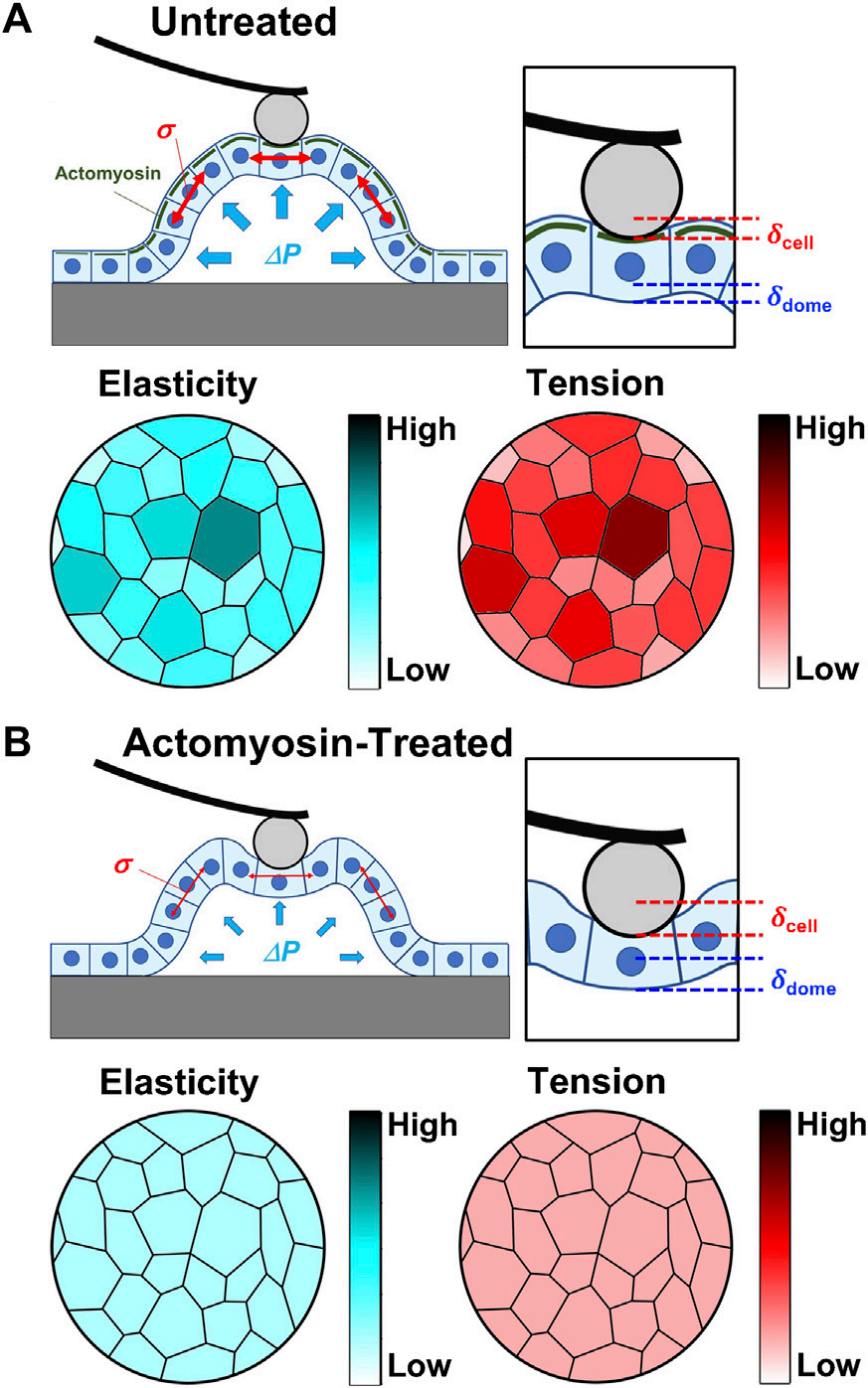
Summary of AFM results and overall hypothesis. Schematic representation of the mechanical properties of domes in untreated **(A)** and actomyosin inhibitor-treated **(B)** conditions. In untreated conditions, actomyosin (green) was accumulated into cell cortical regions in domes cells compared with the surrounding monolayers. The intercellular tension *σ* at a high value was balanced with the internal pressure Δ*P*, where the elasticity and tension of single cells increased with cell stretching. As actomyosin structures were disrupted in domes, the intercellular tension *σ* attained a low value that was balanced with the internal pressure Δ*P*, while the cell stiffness and tension had no apical cell area dependence.

Spontaneous formation of epithelial domes initially occurs due to fluid accumulation between the cell monolayer and substrate, which is a result of unidirectional epithelial transport in an apicobasal direction (Leighton et al., 1969; Tanner et al., 1983; Ishida-Ishihara et al., 2020). The fluid accumulation peels off from the substrate and increases the fluid-filled luminal volume by increasing the luminal pressure. Our AFM measurements showed that the apical cell area significantly increased in domes compared with monolayer samples (Figure 1E), which is consistent with previous optical microscopic observations (Latorre et al., 2018). During dome formation, the number of cells in the domes almost remains unchanged (Latorre et al., 2018). Therefore, the cells forming the domes were stretched, and the dome tension σ balances the osmotic pressure Δ*P* in an equilibrium state (Figure 5A).

Based on the AFM force–indentation curves (Eq. 2), we defined two indentations resulting in cell and dome deformations (Figure 5A). The cell deformation was attributed to cell stiffness, that is, the apparent Young’s modulus *E*. The dome formation was attributed to the tension and osmotic pressure in the dome structure, which was quantified using *B*; *B* increased with decreasing tension and osmotic pressure and was zero for cases with no deformation (Eq. 2). Since the monolayer tightly adhered to the substrate, the deformation of the multicellular system can be ignored. AFM in monolayer revealed that *B* was almost zero, implying that no sample deformation occurred. Since the myosin activity in domes was inhibited, we observed an increase in *B*, suggesting that the dome tension and osmotic pressure decreased (Figure 2E). These results were consistent with previously reported studies (Latorre et al., 2018).

The apical cell area in the dome *S* was ∼80% larger than that in the monolayer (Figure 1E). According to epithelial model simulation (Latorre et al., 2018), the surface tension of cells in the dome monotonically increased until the dome nominal areal strain was ∼100%. The observations made using AFM showed that *B* was negatively correlated with *S*, thereby supporting the simulation results and suggesting that the dome tension increased when the cells were stretched. This correlation disappeared after depolymerization of actin filaments (Figure 3D) or inhibition of myosin activity (Figure 3F). This indicates that the dome tension was closely associated with the actomyosin structures (Figures 5A, B). The tension of cells in domes was not constant and varied in each dome (Figure 3B), implying that the dome is not in a tension equilibrium state but a frustrated state. It has been observed that even in untreated conditions, epithelial domes exhibit volume fluctuation of slow swelling or shrinking (Latorre et al., 2018; Ishida-Ishihara et al., 2020). Furthermore, we often observed that the domes were gradually contracted during both the latrunculin A and blebbistatin treatments. A similar dome instability has been observed in response to treatment with Rho kinase inhibitor Y-27632 (Latorre et al., 2018), resulting in the swelling of the epithelial dome. The results indicated that when the balance between tension and osmotic pressure was highly perturbed, the cell system underwent a phase change between contracting and swelling states. The contraction is presumably caused by localized disruption of epithelial integrity (Latorre et al., 2018) and/or a decrease in permeability in the apicobasal direction (Leighton et al., 1969; Tanner et al., 1983; Ishida-Ishihara et al., 2020).

The *E* of cells in domes measured using AFM was strongly associated with actin filaments (Figure 2B), which was a similar result to that observed in single cells (Rotsch et al., 1999; Rotsch and Radmacher, 2000; Cai et al., 2013; Cai et al., 2017) and cell monolayers (Fujii et al., 2019). When a part of monolayers changed to a dome, the *E* of cells in the dome increased (Figure 1G) and was positively correlated with *S* (Figure 3A); this indicates that as cells were stretched, the actin filaments were well organized (Figure 5A) together with increasing actin filament density (Figure 4). Cells in a monolayer can sense the stiffness of adjacent cells and alter their stiffness via junction proteins (Fujii et al., 2019), which is reminiscent of single-cell reinforcement (Vogel and Sheetz, 2006). Such cell stiffening has been observed in monolayers of cells that are smaller than 200 μm^2^ (Nehls et al., 2019), which are similar to cells in domes (Figure 3A); however, this is not observed in larger cells (Fujii et al., 2019). Our AFM results conclude that when cells are stretched in a dome, they possibly regulate their stiffness and tension through the cortical cytoskeleton, thereby enhancing their organization. Our findings provide new insights into the mechanical properties of cells in three-dimensional deformable tissues explored using AFM.

## Data Availability

The original contributions presented in the study are included in the article/Supplementary Material, further inquiries can be directed to the corresponding author.
